# Supplementary material for: Chemical Genetic Screen in Drosophila Germline Uncovers Small Molecule Drugs That Sensitize Stem Cells to Insult-Induced Apoptosis
Source: Cells. 2021 Oct 16;10(10):2771. doi: 10.3390/cells10102771 (PMC8534514; doi:10.3390/cells10102771)
Supplement: Supplementary file 1 [file cells-10-02771-s001.zip › cells-1369404-supplementary.pdf]

Supplementary Figure 1

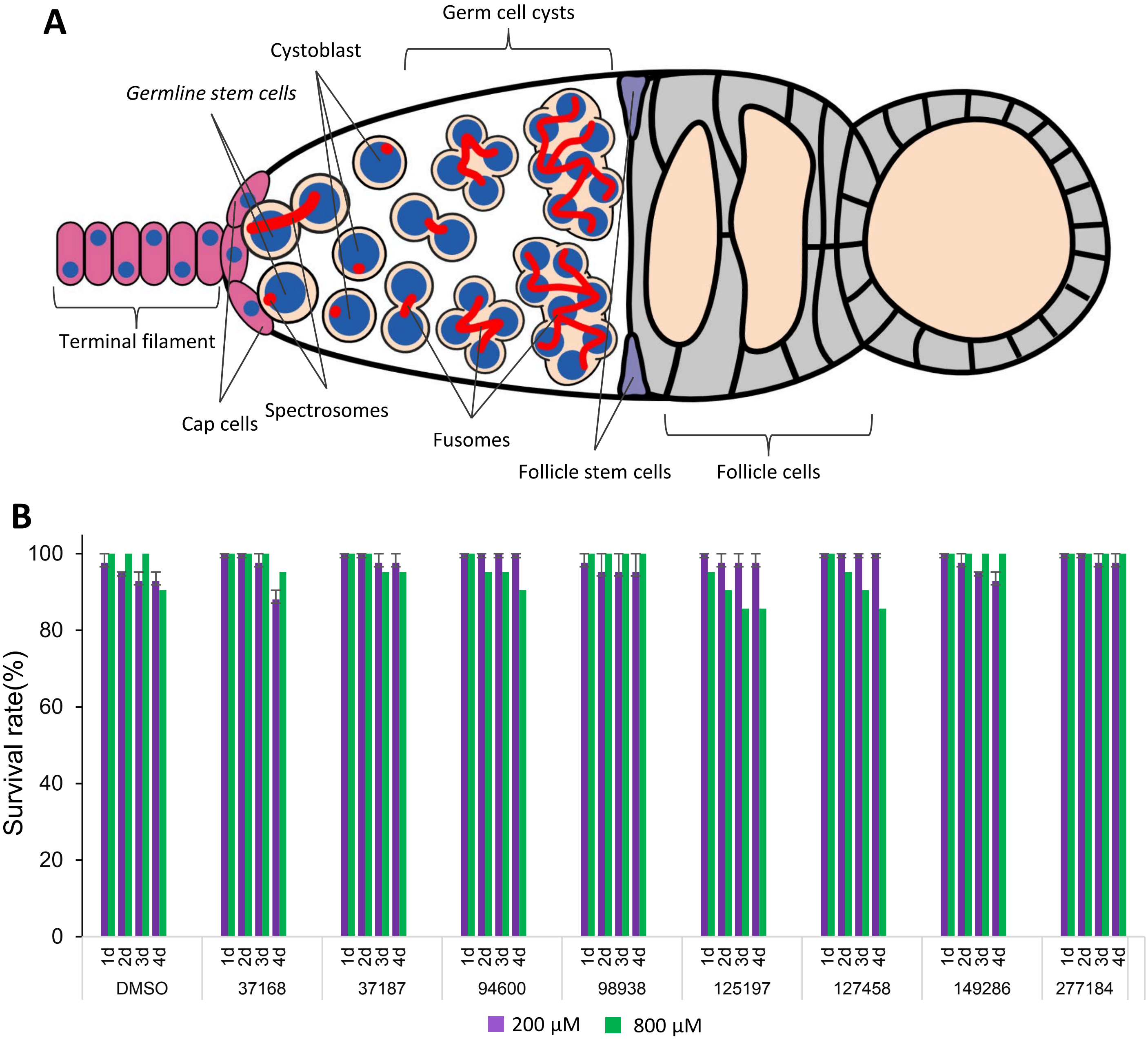

FigureS1- (A) Diagram of the major cell types in the *Drosophila* germarium. At the anterior end, the somatic niche is identified by cap cells, from which the terminal filament cells extend. The cap cells maintain close contact and communicate bidirectionally with the germline stem cells (GSCs). GSCs have a unique, ER-derived organelle, the spectrosome (red), which is usually rounded but elongates during cell division. Canonical GSC asymmetric division reproduces a stem cell and generates a daughter cystoblast. Out of the niche, the cystoblast undergoes four rounds of division with incomplete cytokinesis, giving rise to germ cell cysts (2-, 4-, 8-, or 16-cell). A 16-cell cyst is comprised of the presumptive oocyte and 15 nurse cells. Follicle stem cells divide to encapsulate the 16-cell cyst in a layer of follicle cells. The follicle layer will go on to comprise the somatic epithelium of the egg. (B) A graph depicting Drosophila (both male and females together) rate of survival of when fed with 200 µM (blue) or 800µM (green) of each of small molecules DMSO, NSC-37168, NSC- 37187, NSC-94600, NSC- 98938, NSC-125197, NSC-127458, NSC-149286, NSC-277184 at 1,2,3 and 4 days time-points.

Supplementary Figure 2

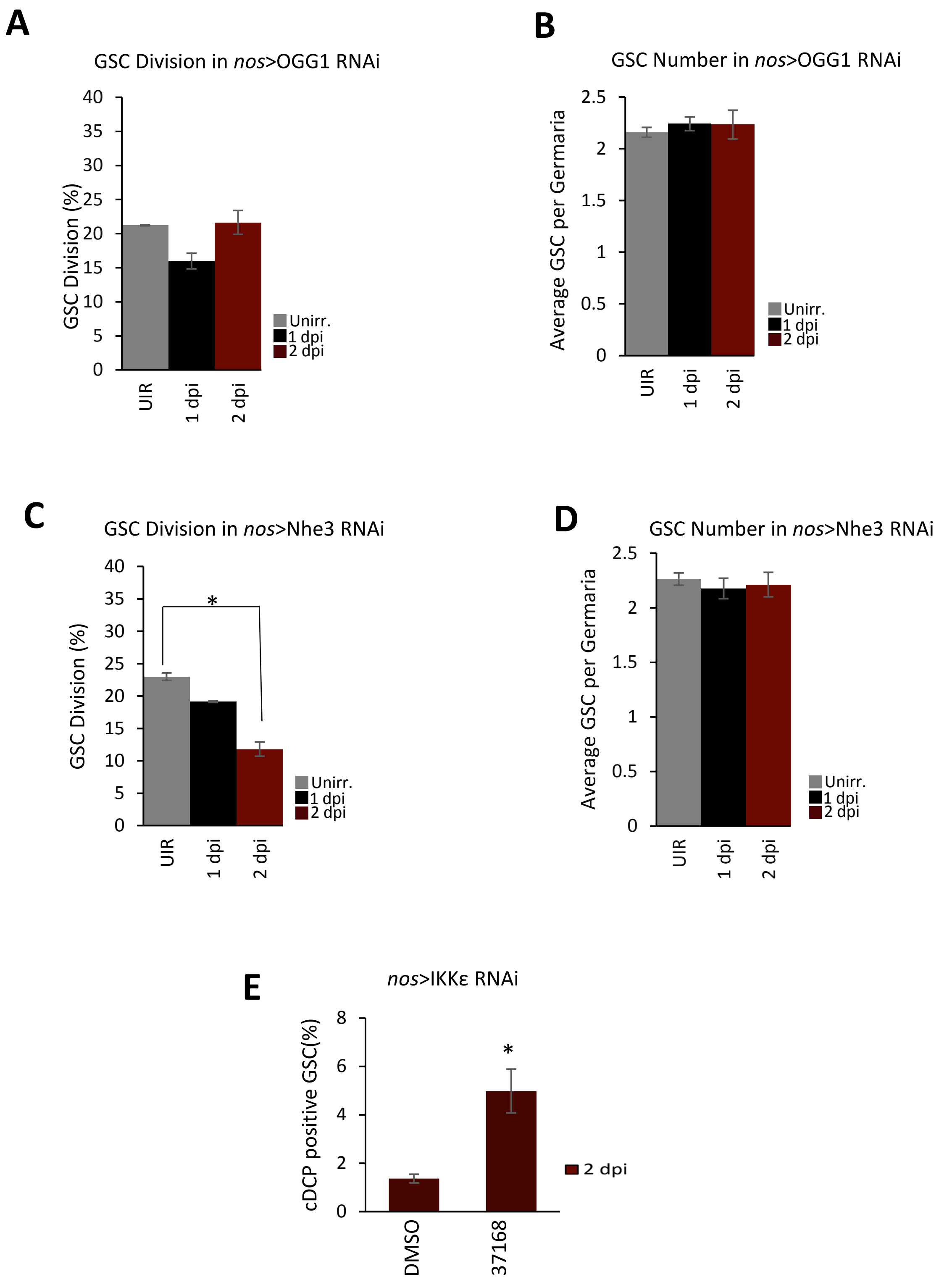

FigureS2- **(A)** Percentage of GSC division upon unirradiation, 1dpi and 2dpi in OGG knockdown.. **(B)** Percentage of GSC division upon unirradiation, 1dpi and 2dpi in Nhe3 knockdown. **(C)** Average GSC number upon unirradiation, 1dpi and 2dpi in OGG knockdown. **(D)** Average GSC number upon unirradiation, 1dpi and 2dpi in Nhoncee3 knockdown. (**E**) Percentage cDCP positive GSCs upon 2dpi in IKKε knockdown upon DMSO and NSC-37168 (200μM) treatment.

Table S1: List of drugs used in the primary drug screening

**NCI number**	cDCP (+/-) male GSCs
NSC- 1451	-
NSC- 1620	-
NSC- 1751	-
NSC- 2561	-
NSC- 2805	-
NSC- 3001	-
NSC- 3961	-
NSC- 4263	-
NSC- 4921	-
NSC- 5995	-
NSC- 6866	-
NSC- 6910	-
NSC- 8090	-
NSC- 8179	-
NSC- 8481	-
NSC- 8813	-
NSC- 9064	-
NSC- 9341	-
NSC- 9358	-
NSC- 9461	-
NSC- 9852	-
NSC- 10091	-
NSC- 10416	-
NSC- 10428	-
NSC- 10995	-
NSC- 11149	-
NSC- 11150	-
NSC- 11826	-
NSC- 11891	-
NSC- 11991	-
NSC- 13151	-

NSC- 13653	-
NSC- 13974	-
NSC- 14303	-
NSC- 14304	-
NSC- 14540	-
NSC- 15362	-
NSC- 15364	-
NSC- 15372	-
NSC- 15571	-
NSC- 16416	-
NSC- 16631	-
NSC- 16813	-
NSC- 17129	-
NSC- 17339	-
NSC- 19063	-
NSC- 19096	-
NSC- 19108	-
NSC- 19115	-
NSC- 19487	-
NSC- 19637	-
NSC- 20045	-
NSC- 20586	-
NSC- 21034	-
NSC- 21678	-
NSC- 21725	-
NSC- 22939	-
NSC- 23123	-
NSC- 23247	-
NSC- 23248	-
NSC- 23672	-
NSC- 23895	-
NSC- 23906	-
NSC- 24035	-

NSC- 25368	-
NSC- 26112	-
NSC- 26113	-
NSC- 26744	-
NSC- 27032	-
NSC- 27628	-
NSC- 28341	-
NSC- 29471	-
NSC- 29620	-
NSC- 30813	-
NSC- 31208	-
NSC- 31664	-
NSC- 31741	-
NSC- 32838	-
NSC- 33005	-
NSC- 33353	-
NSC- 34488	-
NSC- 34769	-
NSC- 34774	-
NSC- 34777	-
NSC- 35676	-
NSC- 35964	-
NSC- 36425	-
NSC- 36520	-
NSC- 36582	-
NSC- 37003	-
NSC- 37168	+
NSC- 47619	-
NSC- 37812	-
NSC- 38042	-
NSC- 38490	-
NSC- 38743	-
NSC- 38845	-

NSC- 38983	-
NSC- 39336	-
NSC- 40383	-
NSC- 40467	-
NSC- 40500	-
NSC- 40614	-
NSC- 40669	-
NSC- 40817	-
NSC- 41092	-
NSC- 41833	-
NSC- 42014	-
NSC- 42212	-
NSC- 42231	-
NSC- 42846	-
NSC- 43308	-
NSC- 44688	-
NSC- 45153	-
NSC- 45291	-
NSC- 46273	-
NSC- 46615	-
NSC- 47496	-
NSC- 47617	-
NSC- 37187	+
NSC- 48422	-
NSC- 48964	-
NSC- 49252	-
NSC- 49652	-
NSC- 49701	-
NSC- 50405	-
NSC- 50572	-
NSC- 50633	-
NSC- 50751	-
NSC- 50858	-

NSC- 51093	-
NSC- 51331	-
NSC- 51936	-
NSC- 53506	-
NSC- 53710	-
NSC- 54834	-
NSC- 55459	-
NSC- 55573	-
NSC- 55770	-
NSC- 55957	-
NSC- 56455	-
NSC- 56906	-
NSC- 56914	-
NSC- 57103	-
NSC- 57165	-
NSC- 57318	-
NSC- 57345	-
NSC- 57670	-
NSC- 57741	-
NSC- 57794	-
NSC- 57890	-
NSC- 58907	-
NSC- 59776	-
NSC- 60034	-
NSC- 60239	-
NSC- 60266	-
NSC- 60373	-
NSC- 60377	-
NSC- 60419	-
NSC- 60530	-
NSC- 60548	-
NSC- 61888	-
NSC- 61910	-

NSC- 62129	-
NSC- 62318	-
NSC- 62511	-
NSC- 62609	-
NSC- 62611	-
NSC- 62665	-
NSC- 62840	-
NSC- 63001	-
NSC- 63311	-
NSC- 63314	-
NSC- 63865	-
NSC- 63963	-
NSC- 64952	-
NSC- 66241	-
NSC- 66837	-
NSC- 67307	-
NSC- 67546	-
NSC- 68657	-
NSC- 68982	-
NSC- 69421	-
NSC- 70534	-
NSC- 70717	-
NSC- 70959	-
NSC- 71795	-
NSC- 72292	-
NSC- 73054	-
NSC- 73170	-
NSC- 73482	-
NSC- 75241	-
NSC- 75585	-
NSC- 75786	-
NSC- 75846	-
NSC- 75885	-

NSC- 77422	-
NSC- 77596	-
NSC- 77913	-
NSC- 78130	-
NSC- 78609	-
NSC- 78999	-
NSC- 79010	-
NSC- 79139	-
NSC- 79253	-
NSC- 79538	-
NSC- 79582	-
NSC- 80141	-
NSC- 80807	-
NSC- 81018	-
NSC- 81120	-
NSC- 81213	-
NSC- 81462	-
NSC- 81660	-
NSC- 81703	-
NSC- 82339	-
NSC- 82769	-
NSC- 83076	-
NSC- 83237	-
NSC- 83339	-
NSC- 83345	-
NSC- 83715	-
NSC- 84200	-
NSC- 85179	-
NSC- 85326	-
NSC- 85331	-
NSC- 87352	-
NSC- 87822	-
NSC- 88811	-

NSC- 88882	-
NSC- 88883	-
NSC- 88962	-
NSC- 88998	-
NSC- 89249	-
NSC- 89258	-
NSC- 89720	-
NSC- 89723	-
NSC- 91438	-
NSC- 91516	-
NSC- 92207	-
NSC- 92264	-
NSC- 92709	-
NSC- 92753	-
NSC- 92794	-
NSC- 93260	-
NSC- 93427	-
NSC- 93817	-
NSC- 94600	+
NSC- 96491	-
NSC- 96979	-
NSC- 97090	-
NSC- 97104	-
NSC- 97538	-
NSC- 98683	-
NSC- 98857	-
NSC- 98938	+
NSC- 99756	-
NSC- 99796	-
NSC- 100120	-
NSC- 100729	-
NSC- 101266	-
NSC- 101653	-

NSC- 101777	-
NSC- 102025	-
NSC- 102086	-
NSC- 102288	-
NSC- 102509	-
NSC- 103770	-
NSC- 103775	-
NSC- 104969	-
NSC- 105827	-
NSC- 106045	-
NSC- 106208	-
NSC- 106261	-
NSC- 106282	-
NSC- 106461	-
NSC- 106506	-
NSC- 106570	-
NSC- 106863	-
NSC- 108235	-
NSC- 108655	-
NSC- 108750	-
NSC- 108753	-
NSC- 108972	-
NSC- 109084	-
NSC- 109086	-
NSC- 109174	-
NSC- 109176	-
NSC- 109231	-
NSC- 109292	-
NSC- 109466	-
NSC- 109528	-
NSC- 109719	-
NSC- 109813	-
NSC- 109885	-

NSC- 111107	-
NSC- 111118	-
NSC- 111552	-
NSC- 112677	-
NSC- 112965	-
NSC- 112975	-
NSC- 113486	-
NSC- 114490	-
NSC- 114831	-
NSC- 116565	-
NSC- 117386	-
NSC- 117554	-
NSC- 117741	-
NSC- 117922	-
NSC- 118723	-
NSC- 118832	-
NSC- 119969	-
NSC- 120286	-
NSC- 120307	-
NSC- 120312	-
NSC- 120844	-
NSC- 121781	-
NSC- 122131	-
NSC- 122280	-
NSC- 122297	-
NSC- 122376	-
NSC- 122987	-
NSC- 123141	-
NSC- 123458	-
NSC- 124146	-
NSC- 125043	-
NSC- 125197	+
NSC- 125727	-

NSC- 126224	-
NSC- 126405	-
NSC- 126757	-
NSC- 127216	-
NSC- 127458	+
NSC- 127947	-
NSC- 128068	-
NSC- 128141	-
NSC- 128737	-
NSC- 128751	-
NSC- 129220	-
NSC- 131982	-
NSC- 134577	-
NSC- 134580	-
NSC- 134784	-
NSC- 134785	-
NSC- 135351	-
NSC- 136065	-
NSC- 138389	-
NSC- 139021	-
NSC- 145180	-
NSC- 147829	-
NSC- 149286	+
NSC- 150982	-
NSC- 151901	-
NSC- 152632	-
NSC- 154316	-
NSC- 154718	-
NSC- 155196	-
NSC- 155698	-
NSC- 155703	-
NSC- 156571	-
NSC- 160005	-

NSC- 162292	-
NSC- 162915	-
NSC- 163104	-
NSC- 163158	-
NSC- 163920	-
NSC- 164965	-
NSC- 166900	-
NSC- 168225	-
NSC- 169458	-
NSC- 169566	-
NSC- 173101	-
NSC- 173103	-
NSC- 176324	-
NSC- 177952	-
NSC- 177989	-
NSC- 191029	-
NSC- 194242	-
NSC- 194243	-
NSC- 195031	-
NSC- 195327	-
NSC- 196515	-
NSC- 203065	-
NSC- 206630	-
NSC- 211490	-
NSC- 227309	-
NSC- 234764	-
NSC- 246415	-
NSC- 269905	-
NSC- 272275	-
NSC- 276369	-
NSC- 277184	+
NSC- 278741	-
NSC- 279834	-

NSC- 281639	-
NSC- 281816	-
NSC- 284701	-
NSC- 287065	-
NSC- 287495	-
NSC- 288686	-
NSC- 292253	-
NSC- 295701	-
NSC- 298197	-
NSC- 299514	-
NSC- 300289	-
NSC- 302584	-
NSC- 302867	-
NSC- 303244	-
NSC- 303603	-
NSC- 303800	-
NSC- 304902	-
NSC- 305798	-
NSC- 311165	-
NSC- 311723	-
NSC- 311727	-
NSC- 317003	-
NSC- 319990	-
NSC- 321484	-
NSC- 322661	-
NSC- 331198	-
NSC- 331208	-
NSC- 335649	-
NSC- 338205	-
NSC- 339578	-
NSC- 341902	-
NSC- 342460	-
NSC- 343256	-

NSC- 344494	-
NSC- 345845	-
NSC- 351110	-
NSC- 361056	-
NSC- 365560	-
NSC- 366808	-
NSC- 370387	-
NSC- 372063	-
NSC- 400770	-
NSC- 407282	-
NSC- 513815	-
NSC- 637153	-
NSC- 650438	-
NSC- 664971	-
NSC- 672441	-
NSC- 375105	-
NSC- 13785	-
NSC- 12644	-
NSC- 12646	-
NSC- 14311	-
NSC- 14380	-
NSC- 13248	-
NSC- 13345	-
NSC- 15358	-
NSC- 13579	-
NSC- 15359	-
NSC- 44750	-
NSC- 46492	-
NSC- 13658	-
NSC- 37433	-
NSC- 135412	-
NSC- 665497	-
NSC- 321506	-

NSC- 11624	-
NSC- 131388	-
NSC- 133195	-
NSC- 140892	-
NSC- 408734	-
NSC- 379651	-
NSC- 36693	-
NSC- 46492	-
NSC- 48388	-
NSC- 127468	-

Table S2: Raw and corrected average absorbance data for alamarBlue assay of MCF7 cells monolayer

Raw Average Absorbance

	1 hr	24 hr	48 hr
DMSO	184633	540111.333	543782.667
Rapa, 10uM	167327	539765.667	542561.333
Ellipticine (71795), 10uM	163477.5	538714	543699
Camptothecin (94600), 10uM	168059	539765.5	547901.5
37168, 10uM	138261.5	543960.5	539237
37187, 10uM	158193.5	541079.5	540551
98938, 10uM	150064	541869	543964
125197, 10uM	162826.5	545536	545801
127458, 10uM	181172.5	540550.5	543439.5
149286, 10uM	164040.5	540553.5	539759.5
277184, 10uM	176380.5	541340.5	544226.5
Triton X-100, 0.02%	31486.6667	86746	146511.333
Triton X-100, 0.1%	14751	69154.6667	128282
[blank]	7248	35831.1667	74930

Corrected Average Absorbance

	1 hr	24 hr	48 hr
DMSO	177385	504280.167	468852.667
Rapa, 10uM	160079	503934.5	467631.333
Ellipticine (71795), 10uM	156229.5	502882.833	468769
Camptothecin (94600), 10uM	160811	503934.333	472971.5
37168, 10uM	131013.5	508129.333	464307
37187, 10uM	150945.5	505248.333	465621
98938, 10uM	142816	506037.833	469034
125197, 10uM	155578.5	509704.833	470871
127458, 10uM	173924.5	504719.333	468509.5
149286, 10uM	156792.5	504722.333	464829.5
277184, 10uM	169132.5	505509.333	469296.5
Triton X-100, 0.02%	24238.6667	50914.8333	71581.3333
Triton X-100, 0.1%	7503	33323.5	53352
[blank]	0	0	0

Absorbance SEM

	1 hr	24 hr	48 hr	Replicates
DMSO	9296.98376	1065.8268	631.481415	3
Rapa, 10uM	14559.9888	3152.167	2575.55795	3
Ellipticine (71795), 10uM	1521.5	1045	2893	2
Camptothecin (94600), 10uM	1797	1051.5	1307.5	2
37168, 10uM	657.5	529.5	1055	2
37187, 10uM	8524.5	1308.5	3420	2
98938, 10uM	5023	1046	5	2
125197, 10uM	8949.5	4202	784	2
127458, 10uM	2461.5	1305.5	3674.5	2
149286, 10uM	5226.5	3944.5	3141.5	2
277184, 10uM	5350.5	2101.5	788.5	2
Triton X-100, 0.02%	1514.07368	5123.79804	6730.27385	3
Triton X-100, 0.1%	870.355674	3604.54548	4784.14991	3
